# Graphs of protein-water hydrogen bond networks to dissect structural movies of ion-transfer microbial rhodopsins

**DOI:** 10.3389/fchem.2022.1075648

**Published:** 2023-01-13

**Authors:** Éva Bertalan, Ana-Nicoleta Bondar

**Affiliations:** ^1^ Physikzentrum, RWTH Aachen University, Aachen, Germany; ^2^ Forschungszentrum Jülich, Institute of Computational Biomedicine, Jülich, Germany; ^3^ Faculty of Physics, University of Bucharest, Măgurele, Romania

**Keywords:** hydrogen-bond networks, TR-SFX, microbial rhodopsin, structural movie, graphs, C-graphs, bridge algorithm

## Abstract

Microbial rhodopsins are membrane proteins that use the energy absorbed by the covalently bound retinal chromophore to initiate reaction cycles resulting in ion transport or signal transduction. Thousands of distinct microbial rhodopsins are known and, for many rhodopsins, three-dimensional structures have been solved with structural biology, including as entire sets of structures solved with serial femtosecond crystallography. This sets the stage for comprehensive studies of large datasets of static protein structures to dissect structural elements that provide functional specificity to the various microbial rhodopsins. A challenge, however, is how to analyze efficiently intra-molecular interactions based on large datasets of static protein structures. Our perspective discusses the usefulness of graph-based approaches to dissect structural movies of microbial rhodopsins solved with time-resolved crystallography.

## Introduction

Microbial rhodopsins belong to a large family of seven-helical membrane proteins in which photo-isomerization of the covalently-bound retinal molecule triggers reaction cycles resulting in ion transfer or photo-sensing (Govorunova et al., 2017). The diversity of biological functions performed by microbial rhodopsins underlines their importance to dissect sequence-structure-function relationships. Moreover, some of the microbial rhodopsins are used as optogenetic tools to control the membrane potential of excitable cells (Zhang et al., 2006; Gunaydin et al., 2010; Berndt et al., 2011; Kandori, 2020). Decades of studies have led to a detailed understanding of the general principles of action of microbial rhodopsins—for comprehensive reviews, see, e.g., refs. (Lanyi, 1999; Heberle, 2000; Balashov and Ebrey, 2001; Herzfeld and Lansing, 2002; Kandori, 2004; Wickstrand et al., 2015; Bondar and Smith, 2017; Govorunova et al., 2017; Kandori, 2020; Brown, 2022). Here, we focus on the usefulness of graph computations to evaluate structural changes along reaction cycles of ion-transfer microbial rhodopsins based on structural movies derived with structural biology.

During its reaction cycle, an ion-transfer microbial rhodopsin undergoes a sequence of protein structural changes that couple to the retinal isomeric state, relocation of discrete internal water molecules, and ion transfer. Time scales for inter-conversions among subsequent intermediate states can vary substantially, for example, in the case of the bacteriorhodopsin (BR) proton pump, the lifetimes of intermediate states that have been characterized with spectroscopy range from the femtoseconds to milliseconds (Lanyi, 1993). Structural biology has provided invaluable data on the architecture and structural dynamics of microbial rhodopsins—from the first electron microscopy structure of BR solved at a resolution of 7 Å (Henderson and Unwin, 1975), to crystal structures solved at resolutions of 1.05–1.6 Å for BR (Borshchevskiy et al., 2022), *Acetabularia* rhodopsin-1 (Furuse et al., 2015), and arachaerhodopsin-3 (Bada Juarez et al., 2021); recently, entire structural movies of structural changes along the reaction cycle, up to the millisecond time domain, were solved with time-resolved serial femtosecond crystallography (TR-SFX) for BR (Nango et al., 2016; Nass et al., 2019; Weinert et al., 2019), the sodium pumping rhodopsin KR2 (Skopintsev et al., 2020), and for channelrhodopsin chimera C1C2 (Oda et al., 2021). For the chloride-ion pumping rhodopsin, CIR, TR-SFX resolved the early-stage dynamics within 100 ns after illumination (Yun et al., 2021).

Data from TR-SFX may be combined into sets of structures for given time intervals, such as an early picosecond time domain, nanosecond-, microsecond-, and millisecond-domains. Together, the ensembles of average protein structures provide an overview of the protein structural dynamics in the crystal environment, which are relevant to reaction cycles in physiological conditions–as verified for BR (Nango et al., 2016) and KR2 (Skopintsev et al., 2020). By providing a high-resolution view of structural rearrangements along key steps of the reaction cycle, datasets of TR-SFX structures are a unique opportunity to dissect the time evolution of protein conformational changes, and to understand how protein conformational changes ultimately lead to ion transfer.

Hydrogen(H)-bond networks are central to formulating hypotheses about reaction mechanisms of membrane transporters in general. In the particular case of ion-transfer microbial rhodopsins, H-bond networks of the retinal Schiff base, and of protein sidechains directly involved in ion transfer, are thought essential for functionality. For microbial rhodopsins whose structural movies have been solved with TR-SFX, the challenge is how to dissect the entire H-bond network of the protein, and to identify sites where H-bonds break or form as the protein passes from one intermediate state to the next. We argue here that graph-based algorithms that compute and compare H-bond networks in datasets of static protein structures enable us to dissect the structural movies captured in experimental data.

**Graphs of H-bond networks computed from static protein structures of microbial rhodopsins**. *An H-bond graph* consists of *nodes*–here, the H-bonding protein groups, and *edges*, which here are direct or water-mediated H-bonds between protein groups. A *local H-bond cluster*, or *local H-bond network*, consists of a subset of nodes and edges that are all interconnected to each other.

Let us consider the structure of the resting state of a microbial rhodopsin, which we label as a reference structure *R*, and two intermediate-state structures, *I1 and I2*. *The conserved H-bond graph* of structures *R*, *I1* and *I2* consists of the nodes (H-bonding groups) and edges (H-bonds) that are common to the three structures within a set conservation threshold (Bertalan et al., 2020; Bertalan et al., 2021). That is, for three static structures of microbial rhodopsins captured at distinct moments of time, the conserved H-bond graph contains the H-bonding protein groups and their H-bonds that remain part of the network. The *difference H-bond graph* of structure *I2* relative to that conserved H-bond graph indicates which H-bonding groups and H-bonds are present in structure *I2*, but not in structures *R* or *I1*. The *comparison H-bond graph* of structures *I2* and *R* indicates H-bonding groups and H-bonds present in both structures, vs. only in *I2/R*.

To compute H-bond graphs we used the graph-based algorithms Bridge (Siemers et al., 2019; Siemers and Bondar, 2021) and C-Graphs (Bertalan et al., 2021) with standard geometric criteria of ≤3.5 Å distance between the H-bond donor and acceptor hetero-atoms; we included water bridges of up to three H-bonded water molecules between protein sidechains. To examine the location of the H-bond networks, protein structures were pre-aligned and their H-bond graphs projected with C-Graphs along the membrane normal (Bertalan et al., 2021). From the projected H-bond graphs we estimated the length of the networks and identified sites where H-bond networks become interrupted or connected in a given structure.

**FIGURE 1:**
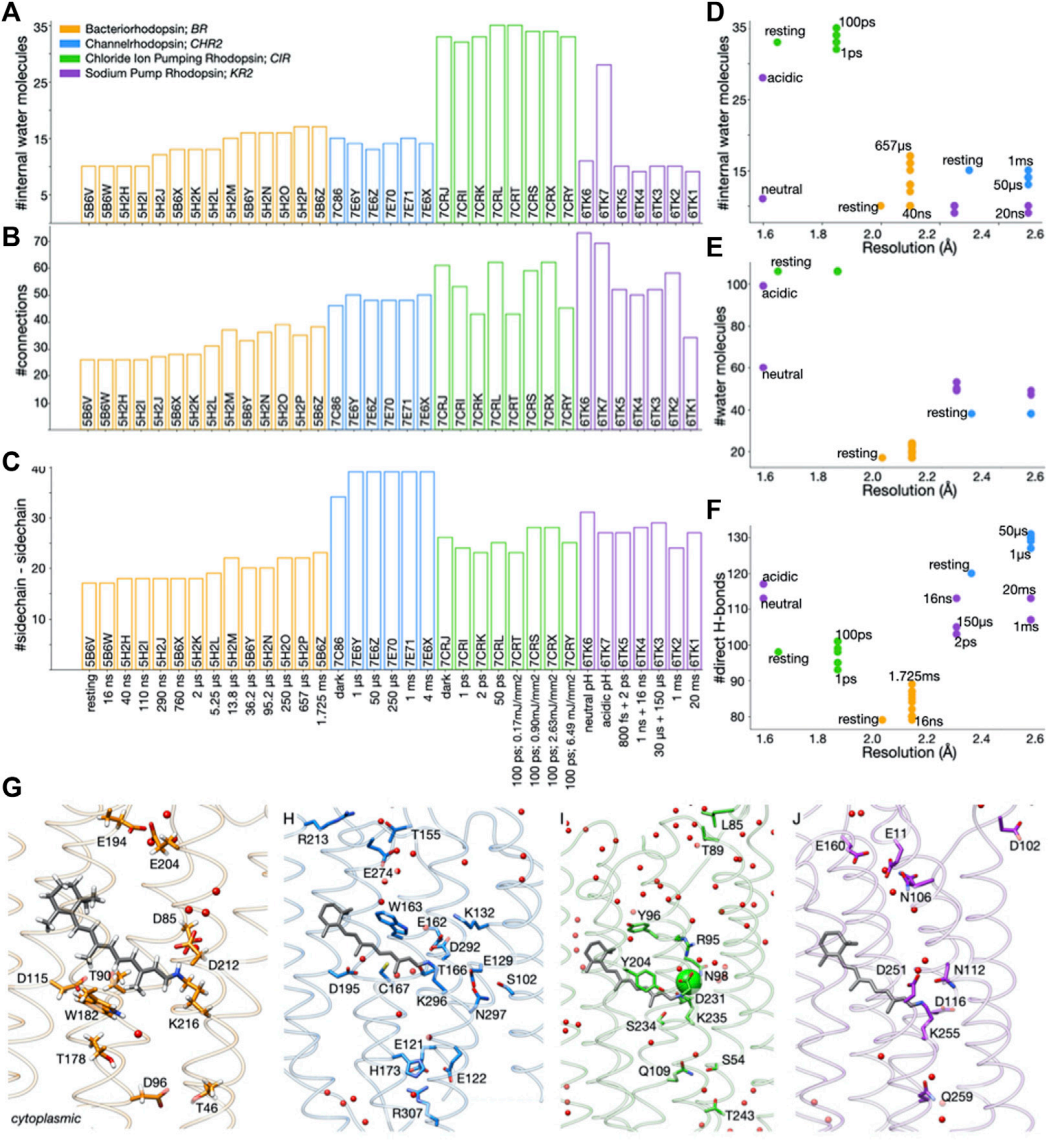
Water-mediated H-bonds in datasets of static structures of microbial rhodopsins. **(A–C)** Number of internal water molecules (panel A), direct and water-mediated H-bond connections **(B)**, and direct sidechain-sidechain H-bonds **(C)**, in datasets of microbial rhodopsin structures: orange, BR structures from 0 ms to 1.725 ms after illumination (Nango et al., 2016); blue, channelrhodopsin (Oda et al., 2021), green, CIR (Yun et al., 2021), magenta, KR2 (Skopintsev et al., 2020). **(D–F)** Number of internal water molecules **(D)**, total number of waters **(E)**, and number of direct H-bonds between sidechains and between sidechains and backbone **(F)** as a function of the resolution at which the structure was solved. In panels D–F, each dot represents a microbial rhodopsin structure, color-coded as in panel **(A) (G–J)** Molecular graphics of BR **(G)**, C1C2 **(H)**, CIR **(I)**, and KR2 **(J)**. Structures were downloaded from the Protein Data Bank, PDB (Berman et al., 2000) and aligned along the membrane normal using Chimera (Pettersen et al., 2004).

We analyzed in total 35 structures of microbial rhodopsins, grouped in 4 datasets according to the corresponding experimental measurement. For each dataset, the resting state is considered as a reference. Protein structures were downloaded from the Protein Data Bank (PDB) (Rose et al., 2021), and the corresponding PDB ID indicated in Figure 1A. To facilitate comparisons of the projections of the H-bond graphs of distinct microbial rhodopsins, we used Chimera (Pettersen et al., 2004) to overlap each structure onto the structure of the dark (resting) state of KR2, PDB ID: 6tk6 (Skopintsev et al., 2020), oriented along the membrane normal with Orientations of Proteins in Membranes, OPM (Lomize et al., 2011).

From the H-bond graphs we extracted the total number of *H-bond connections* between sidechains, which can be direct or water-mediated H-bonds (Figure 1B). Separately, we extracted the number of direct H-bonds between protein sidechains, without water-mediated connections (Figure 1C), and the number of direct H-bonds between protein sidechains and protein backbone groups (Figure 1F). We computed the number of in internal water molecule as the number of water oxygen atoms within the membrane plane indicated by OPM (Lomize et al., 2011).

**Resolution of the structure and the number of water molecules impact H-bond networks.** Internal water molecules are central to reaction mechanisms of ion-transfer microbial rhodopsins (Gerwert et al., 2014; Tomida et al., 2021) because they may, e.g., impact the relative orientation of the protonated retinal Schiff base and its carboxylic primary proton acceptor (Gat and Sheves, 1993), the energetics of proton transfer reactions (Hayashi and Ohmine, 2000; Bondar et al., 2004), the translocation of sodium ions by KR2 (Suomivuouri et al., 2017), and the opening of CHR2 (Ardevol and Hummer, 2017).

For the dataset of 35 static structures considered here, the overall water content depends somewhat on the resolution (Figure 1D): CIR structures solved at resolutions of 1.65–1.85 Å have 106 water molecules each, and the two KR2 structures solved at 1.6 Å resolution have 60 and, respectively 99 water molecules (Figure 1E). Likewise, the number of internal water molecules depends on the resolution–but also on the protein and time domain. The higher resolution CIR structures for 0 ps–100 ps have 32–35 internal waters each, and BR structures for 0–1.725 ms, 10–17 internal waters each. For comparison, each of the 0–4 ms C1C2 structures has 13–15 internal waters, and each of the 800 fs–20 ms KR2 structures, 9–10 waters (Figures 1A, D).

Within a dataset of TR-SFX structures of the same protein, changes in the number of H-bonds of protein sidechains may indicate structural rearrangements leading to the loss/formation of H-bonds along the reaction coordinate of the protein. Typically, within a dataset, the number of sidechain-sidechain H-bonds (Figure 1C) follows the same trend as the total number of H-bond contacts of that protein’s H-bond graph (Figures 1B, C, F): Each of the BR structures has 17–22 sidechain-sidechain H-bonds (Figure 1C), and 26–39 direct and water-mediated contacts between sidechains (Figure 1B); relative to the resting state, the total number of direct H-bonds increases by 10 in the 1.725 ms structure (Figure 1F). The 3 KR2 structures solved at 2.25 Å resolution for 800fs-150 µs, 1 ns + 16 ns, and 30 μs + 150 μs have 50–52 connections each (Figure 1B), and 27–29 sidechain-sidechain contacts (Figure 1C); the 1 ms and 20 ms structures (2.5 Å resolution) have similar numbers of sidechain-sidechain H-bonds (Figure 1C) and internal waters (Figure 1A), but noticeably different numbers of connections (Figure 1B), suggesting rearrangements in water-mediated bridges and/or sidechain-backbone contacts; though distinguished by 17 internal water molecules (Figure 1E), the two 1.6 Å resolution KR2 structures solved at acidic vs. neutral pH have rather similar numbers of H-bond connections (Figure 1B), suggesting rearrangements of the protein-water H-bond network.

The precise contribution that net loss of gain of H-bond connections might bring to the energy profile along the reaction coordinate is unclear. A rough estimation could be made based on double-mutant cycle analyses of BR indicating that, on the average, most sidechain-sidechain H-bonds contribute about 0.6 kcal/mol to the stability of the protein (Joh et al., 2008). Such energetic penalties would be compatible with the energy profile of the first half of the reaction cycle of BR (Bondar et al., 2004; Bondar et al., 2005; Bondar et al., 2007).

Below we inspect closely H-bond graphs of four microbial rhodopsins. For clarity, for the comparison with conserved H-bond graphs we selected four time points representing structural changes at distinct time domains captured with experiments.

**FIGURE 2:**
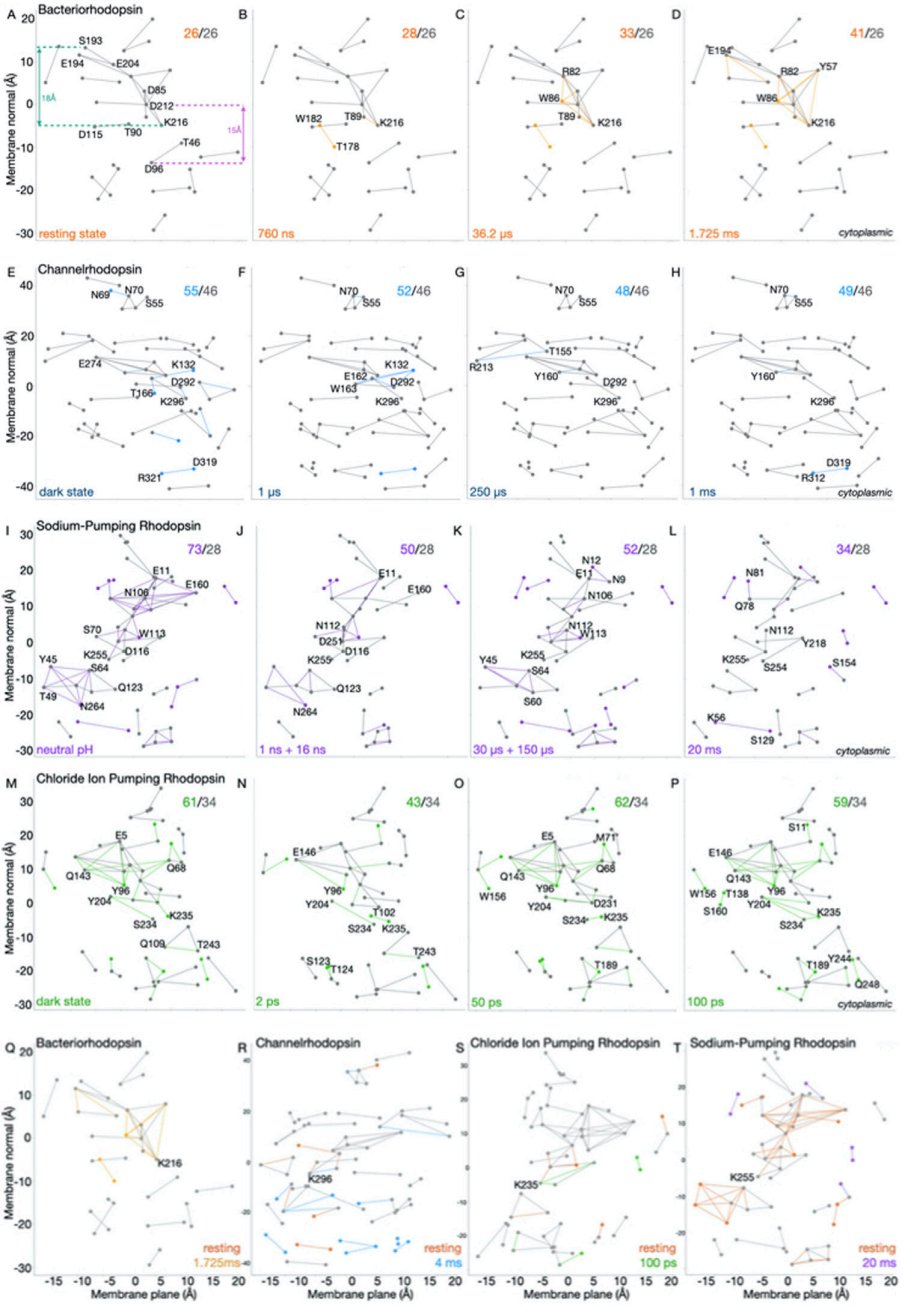
Time evolution of H-bond graphs of microbial rhodopsins. The vertical axis shows the projection along the membrane normal (*z* coordinate) of the Cα atoms of amino acid residues part of the H-bond graphs; the horizontal axis shows the Principal Component Analysis (PCA) projection along the membrane plane. **(A–P)** Difference H-bond graphs. Conserved H-bond graphs computed using Bridge (Siemers et al., 2019; Siemers and Bondar, 2021) within C-Graphs (Bertalan et al., 2021) for BR (panels A–D), C1C2 **(E–H)**, KR2 **(I–L)**, and CIR structures **(M–P)** indicated in Figure 1A are compared to selected structures of each dataset. Nodes and edges colored gray that are present in all structures of the corresponding dataset; different colors indicate nodes and edges present only in the corresponding structure, and numbers in the right upper corner indicate the total/conserved connections. **(Q–T)** Comparison H-bond graphs computed for selected pairs of strructures of BR (Q), C1C2 (R), CIR (S), and KR2 (T).

**H-bond connections at the extracellular side of BR become more extended in the 36.2μs-1.725 ms time domain**. The conserved H-bond graph computed for the 13 BR structures (Nango et al., 2016) has 26 H-bonds (Figures 2A–D). BR resting state hosts an H-bond network with a linear length of ∼18 Å, which includes the primary proton donor (the protonated retinal Schiff base-K216), the primary proton acceptor D85 and D212 (also implicated in proton transfer), and the extracellular proton release dyad E194/E204 (Brown et al., 1995; Dioumaev et al., 1999; Bondar et al., 2004; Phatak et al., 2009); the cytoplasmic proton donor D96, which H-bonds with T46, is within ∼15 Å distance (Cα-Cα) from D85/D212 (Figure 2A) –comparable with distances of ∼10–13 Å between proton-transfer sites in unrelated proton transporters (Bondar, 2022); such distances could be bridged by 3–4 H-bonded water molecules (Bondar, 2022).

Progressive changes in the H-bond connections are observed in the H-bond graphs of the later intermediates. A water-mediated bridge between T178 and W182 appears at 760 ns (Figure 2B) and remains present in the 36.2 µs (Figure 2C) and 1.725 ms structures (Figure 2D). These two latter structures add to the extracellular H-bond network several water-mediated bridges such that this network, though with about the same length of the projection, has greater connectivity than in the resting state (Figures 2A, C, D)—as also indicated by the comparison H-bond graph between the resting and 1.725 ms structures (Figure 2Q). Taken together, the H-bond graphs for the structural movie of BR (Figures 2A–D, Q) indicate that, within ∼1.7 ms, the internal H-bond network that couples D85 to E194/E204 gains connections without extending along the membrane normal.

**C1C2 looses H-bonds in the central protein core**. TR-SFX structures of the *C1C2 channelrhodopsin chimera* solved at a resolution of 2.3 Å captured C1C2 in the dark state and at 1 µs, 50 µs, 250 µs, 1 ms, and 4 ms after illumination (Oda et al., 2021). The conserved H-bond graph computed for all C1C2 structures has 46 H-bonds. Relative to the conserved H-bond graph common to all C1C2 structures, the resting state and the 1 μs structures contain a handful more H-bonds. The H-bond graph of the resting state (Figure 2E) has additional H-bonds at the extracellular network of the protonated retinal Schiff base (K296, see also Figure 1H). H-bonding between the retinal Schiff base and the primary proton acceptor D292, and between E162 and T166, is present only in the resting state (Figure 1H, Figure 2E). The 250 μs structure has additional H-bonds at the extracellular side (see T155, Y160, and R213 in Figure 2G); the 1 ms structure has two additional H-bonds (R312-D319 and S55-N70 in Figure 2H). Overall, unlike the rather localized changes in BR (Figure 2Q), the C1C2 resting vs. 4 ms structures are distinguished by H-bond connections throughout much of the protein (Figure 2R).

**An extensive cytoplasmic H-bond network of KR2 shrinks within 20 ms**. The KR2 sodium pump is of interest for optogenetics applications for the control of neuronal activity (Kato et al., 2015). KR2 couples sodium transport with changes in the protonation of the retinal Schiff base and D116: the Schiff base proton is transferred to D116 and then, following transfer of the sodium ion, back to the retinal Schiff base (Kato et al., 2015). N106, N112, E160, and D251 (Figure 1J) are part of the sodium conductance path (Kato et al., 2015; Skopintsev et al., 2020).

The conserved H-bond graph for the seven KR2 structures of the dataset (Figure 1A) has 28 H-bonds (Figures 2I–L); in all structures, an H-bond network extends from Y45 some ∼12 Å further to the cytoplasmic side, to N264 (Figures 2M–P). In the resting state, this H-bond network includes 14 H-bonds (Figure 2I), of which 10 are lost in the 20 ms structure (Figure 2L). The difference H-bond graph between the resting and 20 ms structures (Figure 2T) indicates loss of H-bonds in the latter, particularly at the cytoplasmic H-bond network of N264, and at the extracellular network of the retinal Schiff base (Figures 2I, T).

**Extensive H-bond rearrangements of the CIR within 200 ps**. In the *Non-labens marinus* chloride pump CIR, the BR proton transfer groups D85 and D96 (Figure 1G) are replaced by N98 and Q109 (Figure 1I) (Yun et al., 2021); BR T89 (Figure 2B), which can function as an intermediate carrier for the Schiff base proton (Bondar et al., 2004; Bondar et al., 2008), is conserved as T102 (Figure 2N). TR-SFX structures of the CIR captured by TR-SFX for the dark, resting state, and for 1 ps, 2 ps, 50 ps, and 200 ps after illumination, suggested rapid structural perturbation such that the chloride ion, which is close to the protonated Schiff base (K235) in the CIR resting state (Figure 1I), is close T102 at 50 ps (Yun et al., 2021). Rapid signal propagation might be needed to ensure an inter-helical pore opens to allow the chloride ion to pass (Yun et al., 2021), and is compatible with the flexible opening of an inter-helical passage observed previously for halorhodopsin (Gruia et al., 2005).

H-bond graphs computed for the eight CIR structures (Figure 1A) have in common 34 H-bonds, but the number of H-bond connections of each of the four structures of the resting, 1 ps, 2 ps, and 50 ps states ranges from 43 to 62 (Figure 1B), and difference H-bond graphs for intermediate states indicate extended H-bond changes throughout the protein (Figures 2M–P). For the 100 ps state, a cross-validation of the signals was interpreted to suggest consistent conformational changes at four different power levels of the laser (Yun et al., 2021). The H-bond graph computations here indicate that the total number of sidechain-sidechain and water-mediated H-bonds between sidechains varies, among the 4 distinct 100 ps structures solved at different laser power levels, between 43 and 62, which is the same interval found for the 0–50 ps structures (Figure 1B). This suggests that structural rearrangements of the CIR H-bond network might depend on the laser power–which could also affect the interpretation of the other structures of the CIR dataset.

## Conclusion

Time-resolved coordinate snapshots of microbial rhodopsins provide invaluable information about the structural rearrangements along the reaction path. Discrete water molecules captured in the structures mediate internal H-bond networks that ensure conformational couplings between remote regions of the protein, and participate in ion transfer reactions.

The graph computations suggest common features in the propagation of structural changes *via* H-bonds and H-bond networks of microbial rhodopsins, but also important differences that could be related to function. Thus, the BR resting and ms-intermediate states are distinguished by connections within an H-bond network that extends through ∼18 Å at the extracellular side, which becomes more inter-connected in the ms structure (Figure 2A). A similar number of H-bond connections distinguishes the internal H-bond network of the resting vs. the 4 ms C1C2 structures but, unlike BR, C1C2 gains H-bonds at the cytoplasmic side (Figure 2R). By contrast, the resting state of KR2 has more extended H-bond connections than the 20 ms structure, particularly at the extracellular and central H-bond clusters (Figure 2T).

A caveat of the analyses of H-bond graphs based on TR-SFX structural movies is that each structure of the data set might represent mixtures of intermediate states (Yun et al., 2021; Barends et al., 2022). Moreover, resolution impacts the overall picture of the internal H-bond networks (Figure 1). We anticipate that future methodological developments in structural biology will allow for more complete structural movies of microbial rhodopsins to be solved at high resolution, and that graph analyses as presented here could be used for an automated assessment of the H-bond fingerprints of intermediate states of microbial rhodopsins.

## Data Availability

The original contributions presented in the study are included in the article/Supplementary Material, further inquiries can be directed to the corresponding author.

## References

[B1] Ardevol A. Hummer G. (2017). Retinal isomerization and water-pore formation in channelrhodopsin-2. Proc. Natl. Acad. Sci. 115, 3557–3562. 10.1073/pnas.1700091115 PMC5889620 29555736

[B2] Bada Juarez J. F. Judge P. J. Adam S. Axford D. Vinals J. Birch J. (2021). Structures of the archaerhodopsin-3 transporter reveal that disordering of internal water networks underpins receptor sensitization. Nat. Comm. 12, 629. 10.1038/s41467-020-20596-0 PMC7840839 33504778

[B3] Balashov S. P. Ebrey T. G. (2001). Trapping and spectroscopic identification of the photointermediates of bacteriorhodopsin at low temperatures¶. Photochem. Photobiol. 73, 453–462. 10.1562/0031-8655(2001)0730453TASIOT2.0.CO2 11367564

[B4] Barends T. R. M. Stauch B. Cherezov V. Schlichting I. (2022). Serial femtosecond crystallography. Nat. Rev. Methods Prim. 2, 59. 10.1038/s43586-022-00141-7 PMC9833121 36643971

[B5] Berman H. M. Westbrook J. Feng G. Gilliland G. Bhat T. N. Weissig H. (2000). The protein Data Bank. Nucleic Acid Res. 28, 235–242. 10.1093/nar/28.1.235 10592235 PMC102472

[B6] Berndt A. Schoeneberger P. Mattis J. Tye K. M. Deisseroth K. Hegemann P. (2011). High-efficiency channelrhodopsins for fast neuronal stimulation at low light levels. Proc. Natl. Acad. Sci. 108, 7595–7600. 10.1073/pnas.1017210108 21504945 PMC3088623

[B7] Bertalan E. Lesca E. Schertler G. F. X. Bondar A.-N. (2021). C-graphs tool with graphical user interface to dissect conserved hydrogen-bond networks: Applications to visual rhodopsins. J. Chem. Inf. Model. 61, 5692–5707. 10.1021/acs.jcim.1c00827 34670076

[B8] Bertalan É. Lešnik S. Bren U. Bondar A.-N. (2020). Protein-water hydrogen-bond networks of G protein-coupled receptors: Graph-based analyses of static structures and molecular dynamics. J. Struct. Biol. 212, 107634. 10.1016/j.jsb.2020.107634 33007367

[B9] Bondar A.-N. Baudry J. Suhai S. Fischer S. Smith J. C. (2008). Key role of active-site water molecules in bacteriorhodopsin proton-transfer reactions. J. Phys. Chem. B 112, 14729–14741. 10.1021/jp801916f 18973373

[B10] Bondar A.-N. Elstner M. Suhai S. Smith J. C. Fischer S. (2004). Mechanism of primary proton transfer in bacteriorhodopsin. Structure 12, 1281–1288. 10.1016/j.str.2004.04.016 15242604

[B11] Bondar A.-N. (2022). Mechanisms of long-distance allosteric couplings in proton-binding membrane transporters. Adv. Protein Chem. Struct. Biol. 128, 199–239. 10.1016/bs.apcsb.2021.09.002 35034719

[B12] Bondar A.-N. Smith J. C. (2017). Protonation-state coupled conformational dynamics in reaction mechanisms of channel and pump rhodopsins. Photochem. Photobiol. 93, 1336–1344. 10.1111/php.12790 28477350

[B13] Bondar A.-N. Suhai S. Fischer S. Smith J. C. Elstner M. (2007). Suppression of the back proton-transfer from Asp85 to the retinal Schiff base in bacteriorhodopsin: A theoretical analysis of structural elements. J. Struct. Biol. 157, 454–469. 10.1016/j.jsb.2006.10.007 17189704

[B14] Bondar A. N. Fischer S. Suhai S. Smith J. C. (2005). Tuning of retinal twisting in bacteriorhodopsin controls the directionality of the early photocycle steps. J. Phys. Chem. B 109, 14786–14788. 10.1021/jp0531255 16852870

[B15] Borshchevskiy V. Kovalev K. Round E. Efremov R. Astashkin R. Bourenkov G. (2022). True-atomic-resolution insights into the structure and functional role of linear chains and low-barrier hydrogen bonds in proteins. Nat. Struct. Mol. Biol. 29, 440–450. 10.1038/s41594-022-00762-2 35484235

[B16] Brown L. S. (2022). Light-driven proton transfers and proton transport by microbial rhodopsins - a biophysical perspective. BBA - Biomembr. 1864, 183867. 10.1016/j.bbamem.2022.183867 35051382

[B17] Brown L. S. Sasaki J. Kandori H. Maeda A. Needleman R. Lanyi J. K. (1995). Glutamic acid 204 is the terminal proton release group at the extracellular surface of bacteriorhodopsin. J. Biol. Chem. 270, 27122–27126. 10.1074/jbc.270.45.27122 7592966

[B18] Dioumaev A. K. Brown L. S. Needleman R. Lanyi J. K. (1999). Fourier transform infrared spectra of a late intermediate of the bacteriorhodopsin photocycle suggest transient protonation of asp-212. Biochemistry 38, 10070–10078. 10.1021/bi990873+ 10433714

[B19] Furuse M. Tamogami J. Hosaka T. Kikukawa T. Shinya N. Hato M. (2015). Structural basis for the slow photocycle and late proton release in *Acetabularia* rhodopsin I from the marine plant *Acetabularia acetabulum* . Acta Cryst. D. 71, 2203–2216. 10.1107/S1399004715015722 26527138

[B20] Gat Y. Sheves M. (1993). A mechanism for controlling the pKa of the retinal protonated Schiff base in retinal proteins. A study with model compounds. J. Am. Chem. Soc. 115, 3772–3773. 10.1021/ja00062a052

[B21] Gerwert K. Freier E. Wolf S. (2014). The role of protein-bound water molecules in microbial rhodopsins. Biochim. Biophys. Acta 1837, 606–613. 10.1016/j.bbabio.2013.09.006 24055285

[B22] Govorunova E. G. Sineschchekov O. A. Li H. Spudich E. N. (2017). Microbial rhodopsins: Diversity, mechanisms, and optogenetic applications. Annu. Rev. Biochem. 86, 845–872. 10.1146/annurev-biochem-101910-144233 28301742 PMC5747503

[B23] Gruia A. D. Bondar A.-N. Smith J. C. Fischer S. (2005). Mechanism of a molecular valve in the halorhodopsin chloride pump. Structure 13, 617–627. 10.1016/j.str.2005.01.021 15837200

[B24] Gunaydin L. A. Yizhar O. Berndt A. Sohal V. S. Deisseroth K. Hegemann P. (2010). Ultrafast optogenetic control. Nat. Neurosci. 13, 387–392. 10.1038/nn.2495 20081849

[B25] Hayashi S. Ohmine I. (2000). Proton transfer in bacteriorhodopsin: Structure, excitation, IR spectra, and potential energy surface analyses by an *ab initio* QM/MM method. J. Phys. Chem. B 104, 10678–10691. 10.1021/jp001508r

[B26] Heberle J. (2000). Proton transfer reactions across bacteriorhodopsin and along the membrane. Biochim. Biophys. Acta 1458, 135–147. 10.1016/s0005-2728(00)00064-5 10812029

[B27] Henderson R. Unwin P. N. T. (1975). Three-dimensional model of purple membrane obtained by electron microscopy. Nature 257, 28–32. 10.1038/257028a0 1161000

[B28] Herzfeld J. Lansing J. C. (2002). Magnetic resonance studies of the bacteriorhodopsin pump cycle. Annu. Rev. Biomol. Struct. 31, 73–95. 10.1146/annurev.biophys.31.082901.134233 11988463

[B29] Joh N. H. Min A. Faham S. Whitelegge J. P. Yang D. Woods V. L. Jr. (2008). Modest stabilization by most hydrogen-bonded side-chain interactions in membrane proteins. Nature 453, 1266–1270. 10.1038/nature06977 18500332 PMC2734483

[B30] Kandori H. (2020). Retinal proteins: Photochemistry and optogenetics. Bull. Chem. Soc. Jpn. 93, 76–85. 10.1246/bcsj.20190292

[B31] Kandori H. (2004). Role of internal water molecules in bacteriorhodopsin. Biochim. Biophys. Acta 1460, 177–191. 10.1016/S0005-2728(00)00138-9 10984599

[B32] Kato H. E. Inoue K. Abe-Yoshizumi R. Kato Y. Ono H. Konno M. (2015). Structural basis for Na^+^ transport mechanism by a light-driven Na^+^ pump. Nature 521, 48–53. 10.1038/nature14322 25849775

[B33] Lanyi J. K. (1999). Bacteriorhodopsin. Intern. Rev. Cytol. 187, 161–202. 10212980 10.1016/s0074-7696(08)62418-3

[B34] Lanyi J. K. (1993). Proton translocation mechanism and energetics in the light-driven pump bacteriorhodopsin. Biochimica Biophysica Acta 1183, 241–261. 10.1016/0005-2728(93)90226-6 8268193

[B35] Lomize M. Pogozheva I. D. Joo H. Mosberg H. I. Lomize A. L. (2011). OPM database and PPM web server: Resources for positioning of proteins in membranes. Nucleic Acid Res. 40, D370–D376. 10.1093/nar/gkr703 21890895 PMC3245162

[B36] Nango E. Royant A. Kubo M. Nakane T. Wickstrand C. Kimura T. (2016). A three-dimensional movie of structural changes in bacteriorhodopsin. Science 354, 1552–1557. 10.1126/science.aah3497 28008064

[B37] Nass G. N. Colletier J.-P. Grünbein M. L. Yang Y. Stensitzki T. Batyuk A. (2019). Three-dimensional view of utrafast dynamics in photoexcited bacteriorhodopsin. Nat. Comm. 10, 3177. 10.1038/s41467-019-10758-0 PMC6639342 31320619

[B38] Oda K. Nomura T. Nakane T. Yamashita K. Inoue K. Ito S. (2021). Time-resolved serial femtosecond crystallography reveals early structural changes in channelrhodopsin. eLife 10, e62389. 10.7554/eLife.62389 33752801 PMC7987342

[B39] Pettersen E. F. Goddard T. D. Huang C. C. Couch G. S. Greenblatt D. M. Meng E. C. (2004). UCSF Chimera - a vizualization system for exploratory research and analysis. J. Comput. Chem. 25, 1605–1612. 10.1002/jcc.20084 15264254

[B40] Phatak P. Frähmcke J. S. Wanko M. Hoffmann M. Strodel P. Smith J. C. (2009). Long-distance proton transfer with a break in the bacteriorhodopsin active site. J. Am. Chem. Soc. 131, 7064–7078. 10.1021/ja809767v 19405533 PMC2746972

[B41] Rose Y. Duarte J. M. Lowe R. Segura J. Bi C. Bhikadiya C. (2021). RCSB Protein Data Bank: Architectural advances towards integrated searching and efficient access to macromolecular structure data from the PDB archive. J. Mol. Biol. 433, 166704. 10.1016/j.jmb.2020.11.003 33186584 PMC9093041

[B42] Siemers M. Bondar A.-N. (2021). Interactive interface for graph-based analyses of dynamic H-bond networks: Application to spike protein S. J. Chem. Inf. Model. 61, 2998–3014. 10.1021/acs.jcim.1c00306 34133162

[B43] Siemers M. Lazaratos M. Karathanou K. Guerra F. Brown L. S. Bondar A.-N. (2019). Bridge: A graph-based algorithm to analyze dynamic H-bond networks in membrane proteins. J. Chem. Theory Comput. 15, 6781–6798. 10.1021/acs/jctc.9b00697 31652399

[B44] Skopintsev P. Ehrenberg D. Weinert T. James D. Kar R. K. Johnson P. J. M. (2020). Femtosecond-to-millisecond structural changes in a light-driven sodium pump. Nature 583, 314–318. 10.1038/s41586-020-2307-8 32499654

[B45] Suomivuouri C.-M. Gamiz-Hernandez A. P. Sundholm D. Kaila V. R. I. (2017). Energetics and dynamics of a light-driven sodium-pumping rhodopsin. Proc. Natl. Acad. Sci. 114, 7043–7048. 10.1073/pnas.1703625114 28611220 PMC5502629

[B46] Tomida S. Kitagawa S. Kandori H. Furutani Y. (2021). Inverse hydrogen-bonding change between the protonated retinal Schiff base and water molecules upon photoisomerization in heliorhodopsin 48C12. J. Phys. Chem. B 125, 8331–8341. 10.1021/acs.jpcb.1c01907 34292728

[B47] Weinert T. Skopintsev P. James D. Dworkowski F. Panepucci E. Kekilli D. (2019). Proton uptake mechanism in bacteriorhodopsin captured by serial synchrotron crystallography. Science 365, 61–65. 10.1126/science.aaw8634 31273117

[B48] Wickstrand C. Dods R. Royant A. Neutze R. (2015). Bacteriorhodopsin: Would the real structural intermediates please stand up? Biochim. Biophys. Acta 1850, 536–553. 10.1016/j.bbagen.2014.05.021 24918316

[B49] Yun J.-H. Li X. Yue J. Park J.-H. Jin Z. Li C. (2021). Early-stage dynamics of chloride ion.pumping rhodopsin revealed by a femtosecond X-ray laser. Proc. Natl. Acad. Sci. 118, e2020486118. 10.1073/pnas.2020486118 33753488 PMC8020794

[B50] Zhang F. Wang L.-P. Boyden E. S. Deisseroth K. (2006). Channelrhodopsin-2 and optical control of excitable cells. Nat. Methods 3, 785–792. 10.1038/NMETH936 16990810

